# Novel Gels for Post-Piercing Care: Evaluating the Efficacy of Pranoprofen Formulations in Reducing Inflammation

**DOI:** 10.3390/gels11050334

**Published:** 2025-04-30

**Authors:** Negar Ahmadi, Maria Rincón, Mireia Mallandrich, Joaquim Suñer-Carbó, Lilian Sosa, Mireya Zelaya, Sergio Martinez-Ruiz, Cecilia Cordero, Ana C. Calpena

**Affiliations:** 1 Department of Pharmacy, Pharmaceutical Technology and Physical Chemistry, Faculty of Pharmacy and Food Sciences, University of Barcelona, Av. Joan XXIII 27-31, 08028 Barcelona, Spain; nahmadah7@alumnes.ub.edu (N.A.); jsuner@ub.edu (J.S.-C.); anacalpena@ub.edu (A.C.C.); 2 Department of Materials Science and Physical Chemistry, Faculty of Chemistry, University of Barcelona, C. Martí i Franquès 1-11, 08028 Barcelona, Spain; 3 Institute of Nanoscience and Nanotechnology (IN2UB), University of Barcelona, Diagonal Ave. 645, 08028 Barcelona, Spain; 4 Centro Experimental en Biociencia (CENBIO), Facultad de Ciencias Químicas y Farmacia, Universidad Nacional Autónoma de Honduras (UNAH), Tegucigalpa 11101, Honduras; lilian.sosa@unah.edu.hn; 5 Instituto de Investigaciones en Microbiología (IIM), Facultad de Ciencias, Universidad Nacional Autónoma de Honduras (UNAH), Tegucigalpa 11101, Honduras; 6 Laboratorio de Técnicas Histológicas, Facultad de Ciencias, Universidad Nacional Autónoma de Honduras (UNAH), Tegucigalpa 11101, Honduras; 7 Departament de Bioquímica i Fisiologia, Facultat de Farmàcia i Ciències de l’Alimentació, University of Barcelona, 08028 Barcelona, Spain; sergio_martinez_ruiz@ub.edu (S.M.-R.); ccorderoalday@ub.edu (C.C.)

**Keywords:** topical gels, Nipagin (methyl 4-hydroxybenzoate), Nipasol (propyl 4-hydroxybenzoate), pranoprofen (PF), NSAID, inflammation reduction, HET-CAM test

## Abstract

Mild to moderate pain for a few hours to several days post-piercing is normal, and the pain is usually accompanied by swelling, redness, and warmth due to the inflammatory response. Cool compresses and over-the-counter analgesics (e.g., NSAIDs) can ease mild discomfort. However, oral NSAIDs may have systemic side effects; for this reason, we propose a topical anti-inflammatory approach. Four pranoprofen-loaded gels were created using different gelling agents: Sepigel^®^ 305 (PF-Gel-Sep), Carbopol^®^ 940 (PF-Gel-Car), Pluronic^®^ F-68 (PF-Gel-Plu), and Lutrol^®^ F-127 (PF-Gel-Lut). The gels were assessed for pH, morphology, FT-IR spectroscopy, rheological properties, spreadability, swelling and degradation, drug release kinetics, skin permeation (cow and human skin), irritation potential (HET-CAM assay), and impact on skin barrier function (TEWL and SCH). The gels exhibited varied rheological properties with PF-Gel-Car showing high viscosity and PF-Gel-Plu very low viscosity. All gels had similar spreadability with PF-Gel-Lut showing the highest. PF-Gel-Car showed the highest amounts of PF released, whereas PF-Gel-Plu led to the highest amount of pranoprofen retained in human and bovine skin. The HET-CAM assay indicated that none of the PF-Gels were irritating. Additionally, PF-Gel-Car and PF-Gel-Plu showed no cytotoxic effects on HaCaT cells. In vivo testing on mice showed that PF-Gel-Car prevented inflammation, while the rest of the gels were able to revert it in 25 min. Skin tolerance tests revealed the gels did not affect TEWL, and some gels improved SCH. The study successfully formulated and characterized four PF-loaded topical gels with potential to be used as an alternative for treating inflammation from piercings and ear tags.

## 1. Introduction

Ear tagging is a widely adopted practice in livestock management, facilitating animal identification through the use of visual, metal, plastic, or electronic radiofrequency identification (RFID) tags. This method enables the efficient monitoring of animal behavior, administration of antiparasitic treatments, record keeping, and disease surveillance at different stages of the animal’s lifecycle [1]. And also piercing is the process of inserting rings, earrings, and other jewelry into body parts such as the earlobes, nose, eyebrows, navel, nipples, and genitalia. Teenagers and young adults often use body piercings to self-express [2]. Furthermore, according to a French survey, women were more likely than men to have a piercing between the ages of 25 and 34 [3]. The face, nose, and ears are the most frequently perforated visible places; the tongue and navel are semi-visible; and the nipples and perineum, which are not visible, are becoming popular places for body art on both sexes [3,4]. Additionally, ear tagging is widely used for identifying beef cattle [1] and other farm animals. However, it causes damage and discomfort and may result in a painful procedure that induces acute pain and inflammation, which can persist if inadequate hygiene measures are taken [1,5]. Piercing and ear-tagging procedures introduce mechanical trauma that disrupts the integrity of tissue, resulting in localized swelling and redness and pain. Therefore, inflammation is a common and expected part of the initial healing process after a piercing. Pain is also common during the piercing procedure and in the early healing phase [6]. However, the intensity, duration, and cause of pain can vary widely depending on the body site, individual sensitivity, technique used, and aftercare practices. The inflammation reaction is largely dependent on the immune system, whose mediators (including histamines, cytokines, and prostaglandins) act to promote tissue repair [6]. Inflammation is a natural, irritating response and can be a process of healing [7,8,9,10,11]. The type and severity of inflammation can vary significantly depending on the depth and extent of tissue damage, impacting the epidermis, dermis, and subcutaneous tissue. Acute inflammation represents the immediate and short-lived response to the needle insertion for piercing; the acute inflammation is marked by classic signs such as redness, swelling, and pain [12]. The depth of tissue loss, particularly in skin wounds, dictates the inflammatory response and subsequent healing process. Injuries that penetrate deeper into the layers, affecting not just the epidermis but also the dermis and subcutaneous tissue, tend to elicit a more pronounced inflammatory response [7]. Different types of injuries can evoke varying inflammatory responses. Mechanical, thermal, and chemical injuries can activate the body’s immune defenses differently. Acute injuries often provoke a robust inflammatory reaction aimed at repairing the damaged tissues and combating potential infections. Conversely, chronic injuries or conditions may lead to prolonged inflammation, which can be detrimental for the affected tissues [12]. Inflammation becomes problematic when it is excessive, prolonged, or associated with trauma because of friction or pressure on the piercing [12]. Not only does the application of ear tags influence the extent of pain and inflammation, but the type of tag used also plays a significant role [7,8,9,10,11]. Studies have shown that metal tags are associated with more damage compared to polyurethane tags. After comparing alterations seen in calves four weeks after the implantation of both metal and plastic ear tags, Johnston and Edwards (1996) [13,14] discovered that metal tags were more common (47.3%) than plastic tags (1.1%) in causing ear injury, tissue irritation and worsening or prolonging the inflammatory phase [15]. Thus, animal welfare has been shown to decline over time as a result of wound inflammation, according to Wendl et al. [14], in either aesthetic or livestock practices with which veterinarians are chiefly concerned [16]. One facet of management is monitoring animal behavior while avoiding upsetting intervention [17]. According to the European Directive on Animal Welfare on the Farm (EU Council Directive 98/58/EC of 20 July 1998 concerning the protection of animals kept for farming purposes), member states must implement measures to guarantee that farm animals do not endure needless pain, suffering, or harm [14].

Mild to moderate pain for a few hours to several days post-piercing is normal, and the pain is usually accompanied by swelling, redness, and warmth due to the inflammatory response. Cool compresses and over-the-counter analgesics (e.g., NSAIDs) can ease mild discomfort [18,19]. Pranoprofen (PF) is a non-steroidal anti-inflammatory medication and a propionic acid molecule with a tricyclic structure and non-steroidal anti-inflammatory drugs (NSAIDs). Pranoprofen’s primary anti-inflammatory mechanism involves inhibiting cyclooxygenase (COX) activity and preventing the conversion of arachidonic acid (AA) derivatives into endogenous prostaglandins (PGs) [18]. Although the oral administration of NSAIDs is the most common route, it has been questioned because of its potential side effects, such as gastrointestinal (GI), renal, and cardiovascular complications, especially in elderly patients [19]. On the other hand, oral administration of pranoprofen (PF) is limited due to its inappropriate biopharmaceutical profile with a short plasmatic half-life, poor aqueous solubility, and instability in aqueous solutions [20]. Some studies have reported that several topical NSAIDs have comparable levels of efficacy to their oral counterparts. Even with a partial loss of efficiency, topical administration provides additional safety and several benefits [21]. It prevents chemical and enzymatic degradation in the GI tract through first-pass metabolism interactions with other drugs that allow direct application to the affected site at specific concentrations, resulting in fewer side effects and better patient compliance [22]. Modulating the drug’s permeability is required to enhance its effectiveness and lessen its negative effects [23].

The current investigation aims to find treatment modalities for piercing in humans and ear tagging in farm animals using topical biomaterial gels. We propose a new topical formulation by which Pranoprofen may reduce the inflammatory response associated with piercing in humans and ear tagging in farm animals such as cows while investigating the drug’s therapeutic benefits. The physicochemical properties of gel types (PF-Gel-Sep, PF-Gel-Car, PF-Gel-Plu, and PF-Gel-Lut formulations), such as viscosity for appropriate skin application, as well as their rheological behavior, have been assessed. The HET-CAM test, cytotoxicity, and in vitro and in vivo skin permeation studies, as well as the in vivo anti-inflammatory study, tested the safety and efficacy of the gel approach.

## 2. Results and Discussion

### 2.1. Gels Formulation

Researchers are interested in biodegradable synthetic polymers due to their stability, safety, and tunable biodegradability, which can extend drug life and achieve sustained release, reducing administration frequency while maintaining drug efficacy [24]. In this work, we used four gelling agents: Carbomer, Pluronic^®^, Lutrol^®^ and Sepigel^®^ to prepare gel formulations containing pranoprofen. Sepigel^®^ 305 provides a smooth and pleasant texture that is easy to apply and comfortable on the skin [25]; it is a non-ionic emulsifier containing fatty oils and polyacrylamides that is commonly incorporated into gels and gel-based creams. It is characterized by its medium-to-high viscosity, cooling effect, and favorable dermo-cosmetic properties, which contribute to its stability and compatibility with a broad range of formulations. Previous investigations of various medications have shown that formulations using this polymer have good stability. They also permit the integration of both hydrophilic and lipophilic compounds [26].

Carbomer is used as a gelling agent in topical formulations [23,24]; it is biocompatible and safe for the use on skin, it is easy to work with, and it results in gels with desirable properties such as spreadability, consistency, and homogeneity [27,28].

Additionally, Lutrol^®^ F-127 and Pluronic^®^ F-68 are also biocompatible and safe for use on skin. The versatility of Pluronic^®^ F-68 makes it a valuable component in pharmaceutical formulations; it can form micelles and gels in aqueous solutions, depending on temperature and concentration. It is used in a broad range of applications for oral, topical, intranasal, vaginal, rectal, ocular, and parenteral routes [29]. They are composed of a hydrophobic poly (propylene oxide) segment joined on both ends to a hydrophilic poly (ethylene oxide) tail that forms the amphiphilic triblock copolymer known as PEO-PPO-PEO [30,31]. Because of their hydrophilic PEO side chains and hydrophobic PPO central segment, poloxamers are amphiphilic and self-assemble into core–shell micelles with diameters ranging from 10 to 100 nm. These adaptable biomedical polymers are a crucial class because they can be altered depending on the therapeutic cargo. Drug delivery across cell barriers is another efficient use of Pluronics. Increasing concentration and temperature have an impact on the micellar structures’ strong packing, which converts Pluronic^®^ micelles into gelation [32].

Parabens are widely utilized as preservatives in pharmaceutical formulations due to their potent antibacterial and antifungal properties as well as their established safety profile for human use at regulated concentrations [33]. Methylparaben, known as nipagin, is one of the relatively few substances that exhibit fungistatic activity against a wide spectrum of microorganisms [34,35].

### 2.2. Physicochemical Characterization of the Gels

#### 2.2.1. PH and Morphological Analysis

In evaluating the effectiveness of gels for topical delivery, measuring pH is essential. The pH level can significantly influence the stability, efficacy, and compatibility of the gel with the skin. Human skin typically has a slightly acidic pH, around 4.5 to 5.5, which helps maintain its barrier function and microbial balance [36]. On the other hand, animal skin usually exhibits higher pH values; for instance, adults’ cows pH is within the range 4.5–7.6 [37], the dog’s skin pH value is in the range 6.2–7.4 [38], horses present skin pH values about 7–7.4. [39], and cattle’s skin pH is in the range 7–8 [40]. Gels with a pH too far outside this range may cause irritation, disrupt the skin barrier, or impact the activity of active ingredients [41]. Studies have shown that maintaining a formulation’s pH close to that of the skin enhances product safety, improves skin compatibility, and can facilitate the delivery of active compounds. Therefore, monitoring and adjusting the pH of gels intended for topical use is critical for optimal performance [42]. Table 1 shows the pH of the four gels after preparation. PF-Gel-Lut and PF-Gel-Plu showed the highest pH values, near neutral values (7.67 and 7.16, respectively), PF-Gel-Sep showed a pH of 6.23 and PF-Gel-Car exhibited a pH of 5.39. Based on the optimal skin pH values for each species, PF-Gel-Car is more suitable for the use on humans, whereas the rest of the gels may be suitable for animal use [37,38,39,40]. The pH values of PF-Gel-Car and PF-Gel-Sep were similar to those for the formulations prepared with the same polymers containing NLC loading pranoprofen [43].

**Figure 1:**
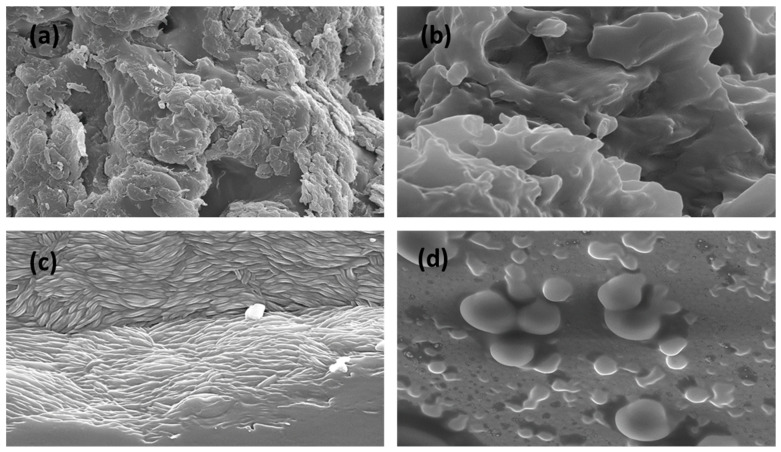
SEM photomicrographs of the dried (**a**) PF-Gel-Car (magnification 4000×); (**b**) PF-Gel-Plu (magnification 15,000×); (**c**) PF-Gel-Lut (magnification 5000×); and (**d**) PF-Gel-Sep (magnification 5000×). Scale bar = 1 µm.

The evaluation of the microstructure of four gels via Scanning Electron Microscopy (SEM) reveals significant information about their behavior in terms of morphology, and such behavior is significant for both functional performance and future use in pharmaceuticals and biomedical materials. The PF-Gel-Car (Figure 1a) exhibited a rough and highly porous structure with non-uniform pores. This type of structure is a sign of high absorption and transportation of the mass structure, and for that reason, it is specifically beneficial for application in high-rate diffusion requirements in wound dressings and in high-rate diffusion requirements in drug delivery systems. High porosity in such a gel can stimulate high-release activity for active ingredients, such as anti-inflammatory drugs, and for that reason, it is specifically beneficial for application in anti-inflammatory and healing-promoting drugs [44]. Porous structures in gels have been previously documented, and it was observed that such structures can have a significant impact on therapeutic activity in drug delivery systems [45]. In contrast, PF-Gel-Plu (Figure 1b) exhibited a denser and smoother morphology, which is reflective of a coherent matrix capable of providing mechanical integrity and sustained delivery. This kind of structure is most beneficial for sustained delivery systems, particularly for long-term use topically, in which the sustained delivery of anti-inflammatory drugs is critical in a manner that extends therapeutic activity. A dense structure can even serve to protect active ingredients from degradation in the environment, which is a consideration that is critical in creating effective transdermal delivery systems [46]. The function of density in a matrix in controlling the delivery of drugs has been emphasized in a variety of studies, citing its role in enhancing transdermal patch efficacy and stability [47]. The PF-Gel-Lut (Figure 1c) exhibited a lamellar or layered structure, suggesting directional crystallization or directional self-assembly during drying, and such a structure can be useful in transdermal patches or a controlled-release system that can deliver drugs in a sustained manner. Layered structures can enhance adhesion in a direction, and such adhesion can enhance anti-inflammatory compounds’ diffusion through dermal tissue, which is important for effective transdermal delivery [48]. Layered structures in drug delivery have been reported to have a key role in delivering drugs, and such structures can maximize therapeutic efficacy [49]. Lastly, PF-Gel-Sep (Figure 1d) showed a dispersed structure with spherical inclusions in a gel matrix, which was most likely a result of phase separation during preparation. Such a structure is potentially used in emulsions in drug delivery or in hydrogels for the encapsulation of drugs with a high level of hydrophobicity. The dispersed nature of the gel could enable the encapsulation and slow delivery of anti-inflammatory drugs with therapeutic delivery for inflamed dermal disease [50]. That dispersed systems can enable the delivery of drugs has been supported by studies implicating efficacy in enhancing the bioavailability of drugs with high hydrophobicity [51].

These structural findings validate such gels’ utility in biomedical applications, including anti-inflammatory drugs for use topically, wound healing, and the long-term delivery of drugs for both immediate and long-term therapeutic activity. Not only are individual types of gels characterized by specific structures at a morphological level, but these in fact have an influence not only over their functional behavior but also over suitability for use in individual pharmaceutical and biomedical applications [52].

#### 2.2.2. Fourier Transform Infrared (FT-IR) Spectroscopy

**Figure 2:**
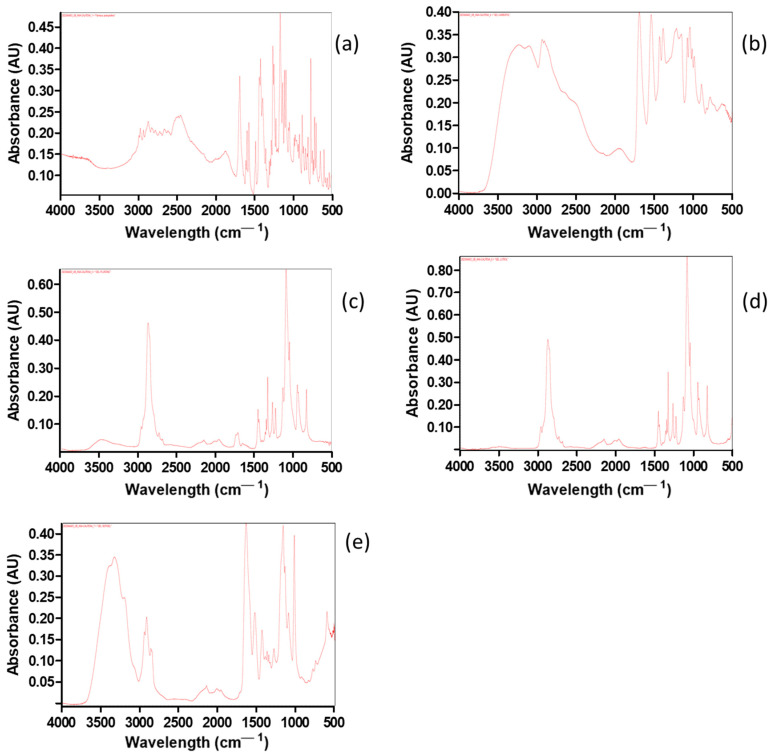
Fourier-transform infrared spectroscopy (FT-IR): (**a**) PF substance; (**b**) PF-Gel-Car, (**c**) PF-Gel-Plu, (**d**) PF-Gel-Lut, and (**e**) PF-Gel-Sep.

Fourier-transform infrared spectroscopy (FT-IR) was conducted to investigate the bonds of the drug formulations and compare them with the reference drug PF. FT-IR spectra of the reference drug and most of the gel formulations, namely, PF-Gels, are depicted in Figure 2a–d. FT-IR spectra show functional groups that exist in each sample and probable interactions between the drug and gel formulations.

Regarding the spectrum of PF-Gel-Car (Figure 2a), the PF (blue) spectrum displays distinctive bands from O-H/N-H stretching vibrations in the 3200–3500 cm^−1^ range. On the other hand, PF-Gel-Car presented a specific fingerprint, especially in the 1500–500 cm^−1^ interval, which evidenced structural differences. However, since the functional groups of the main drug had not varied, no new covalent bond would be formed between the drug and gel PF-Gel-Plu.

Figure 2b shows the bands in the PF (blue) FT-IR spectra that are located at wavenumbers of circa 1700 cm^−1^, corresponding to the stretching vibrations of C=O. These groups belong to the typical chemical structure of pharmaceuticals. In the hydrogen-bonding region, that is, at 3000–3500 cm^−1^, Gel Plu (red) presents lower intensity, meaning fewer O-H/N-H interactions. The fingerprint area (below 1500 cm^−1^) also varies significantly, suggesting that the gel matrix and the medication have different chemical compositions. Figure 2c shows the FT-IR spectra of PF-Gel-Lut; the O-H/N-H stretching zone (3200–3500 cm^−1^) and the C=O stretching region (about 1700 cm^−1^) are where PF (blue) once again displays consistent bands in this comparison. A wide absorption band close to 3000 cm^−1^ is seen in Gel Lut (red), suggesting a distinct hydrogen bonding environment from that of the drug. The distinct structural characteristics of the gel are reflected in the fingerprint area, which exhibits distinct variations. In contrast to the drug’s (blue) thinner bands in this area, the PF-Gel Sep (red) (Figure 2d) has a wide absorption band of about 3200–3400 cm^−1^, indicating strong hydrogen bonding. Significant bands in the fingerprint area (below 1500 cm^−1^) emphasize the structural variations between the medication and gel. There are no apparent new covalent bonds between PF and the gel composition; this also was observed in a previous work of Ahmadi et al. [43] in which pranoprofen was loaded in NLC and these were dispersed in gel formulations composed of Carbomer and Sepigel^®^.

#### 2.2.3. Rheological Measurements of the Gels

**Figure 3:**
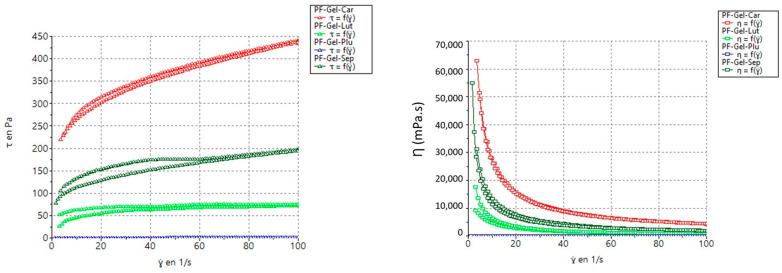
Flow and viscosity curve: PF-Gel-Car, PF-Gel-Plu, PF-Gel-Lut, PF-Gel-Sep.

The rheological properties of the gels were evaluated to determine their flow behavior when forces were applied. PF-Gel-Car has a very high viscosity at 4390.00 ± 14.70 mPa·s, which is indicative of a mostly structured and strong gel. The high yield stress characteristics indicate the development of a highly resistant structure against flow until a critical threshold in shear stress is reached (Figure 3). PF-Gel-Sep exhibits high viscosity at 1964.00 ± 5.16 mPa·s with substantial yield stress, thus providing the right balance between structural stability and ease of use. PF-Gel-Lut presents a reasonably medium viscosity of approximately 742.30 ± 9.32 mPa·s with shear-thinning characteristics, which is ideal for applications that require a smooth flow. Finally, PF-Gel-Plu has a very low viscosity of 18.54 ± 0.13 mPa·s and displays close-on Newtonian behavior. Therefore, it is very suitable for applications when a smooth and easy flow is desired.

The flow curve demonstrates how shear stress (the force per unit area required to move the fluid) varies with the applied shear rate (the rate at which the fluid is deformed) (Table 2, Figure 3). The flow curve of PF-Gel-Lut shows a gradual increase in shear stress with shear rate, which is characteristic of a shear-thinning fluid behavior confirmed by the flow index (*n*) below 1 [53]. This implies that the viscosity decreases with the increase in shear rate, which is a behavior rather common in many gels and suspensions. This property is very useful in applications that require the material to spread out or flow easily when some forces are applied but require a firmer consistency while at rest [54].

The flow curve starts at a high shear stress value even when the shear rate is minimal, indicating that a substantial initial force is needed to initiate flow. Beyond this yield stress, PF-Gel-Car still exhibits shear-thinning behavior. This would imply that such a high yield stress and shear-thinning character shows that a strong internal structure is present in PF-Gel-Car. This can be very important for applications where the product needs to keep its place up to an adequate application of force, such as thick creams or heavy-duty gels [55]. PF-Gel-Sep also exhibits yielding behavior similar to PF-Gel-Car albeit with a lower starting stress. Beyond the yield point, it demonstrates shear-thinning characteristics, making it more workable upon application. This behavior is particularly desirable for applications requiring a balance between flow resistance and ease of spreading, such as in structured liquids or dispersions. The soft yield stress and thixotropic nature of PF-Gel-Sep ensure initial structural integrity while allowing smooth application and spreadability, aligning with findings reported by Ahmadi et al. [43], who observed comparable rheological behavior in pranoprofen-loaded NLC gels with Carbomer and Sepigel^®^ matrices.

PF-Gel-Sep also exhibits yielding like PF-Gel-Car, but the starting stress is small. It is shear thinning beyond the yield point. This behavior is desirable in applications that call upon a degree of flow resistance and at the same time require the product to be easily workable after initial application, such as dispersions or structured liquids, due to its soft yield stress and thixotropy.

**Table 2:** Parameter values of the best-fit rheological model for the tested formulations.

Sample	Model	Consistency (*K*) K (Pa·s)	Flow Index (*n*)	Yield Stress (*τ*0) (Pa)
PF-GEL-LUT	Cross	30.57	0.1949	—
PF-GEL-CAR	Herschel-Bulkley	95.29	0.2805	92.97
PF-GEL-PLU	Cross	0.02242	0.961	0.00418
PF-GEL-SEP	Herschel-Bulkley	21.89	0.3976	58.09

*K*: consistency coefficient [56,57]; *n*: flow behavior index [53].

#### 2.2.4. Spreadability Test

The spreadability test assesses how easily a product can be applied to the skin and spread over a desired area. This test typically involves applying a controlled weight to the formulation and measuring the resulting spread area, which helps in understanding the formulation’s consistency, viscosity, and ease of use [58]. A product’s spreadability is closely related to its user acceptability and efficacy, as formulations that spread evenly ensure better coverage and absorption of active ingredients [59]. Optimizing spreadability is particularly important for gels and creams, where a balance between ease of spread and sufficient adhesion to the skin is crucial [60,61].

**Figure 4:**
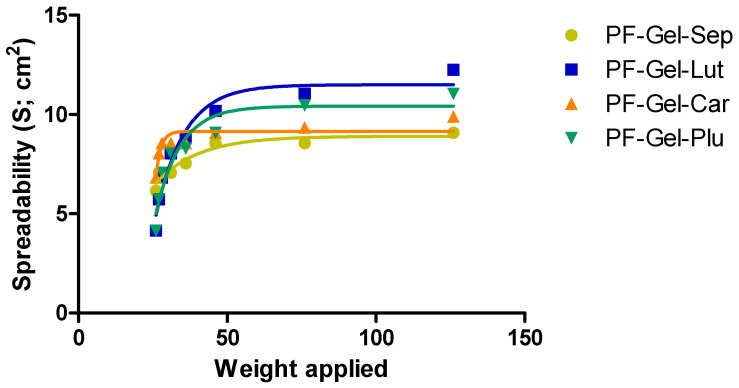
The capacity of spreading for the 4 gels as a function of weight.

All gel formulations showed quite similar maximum spreading values (from 9 to 12 cm^2^ for 126 g applied) (Figure 4), among which PF-Gel-Sep is the lower while PF-Gel-Lut is the higher. When comparing to previous works, Ahmadi et al. [43] tested the spreadability of gel formulation containing pranoprofen loaded in NLC, and the researchers observed a higher spreadability capacity of the gels composed of NLC. High spreadability values indicate that a formulation easily spreads over a large area under applied force, reflecting lower viscosity and a smoother, more spreadable consistency. This is generally favorable for products that need to cover large or sensitive areas, as it allows for an effortless application and even distribution of active ingredients [25]. High spreadability is typically desirable for lotions, creams, and gels where easy application and comfort are priorities.

#### 2.2.5. Swelling and Degradation Tests

**Figure 5:**
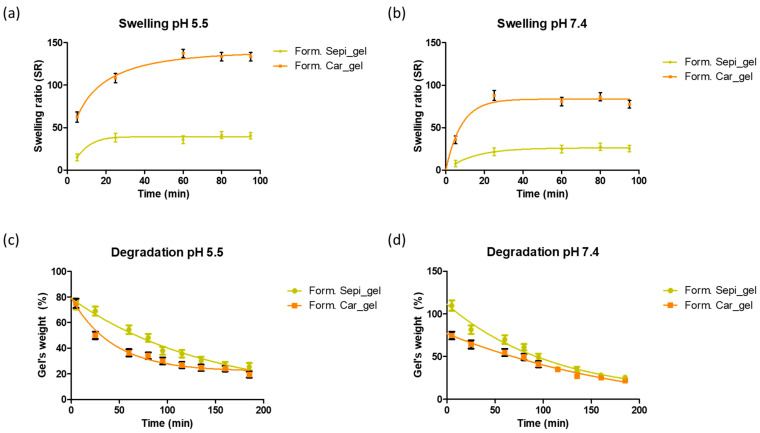
Swelling capacity as solvent uptake capacity over time. (**a**,**b**) Degradation behavior as weight loss over time (**c**,**d**) of PF-Gel-Car and PF-Gel-Sep. The tests were conducted at pH 5.5 and 7.4.

The swelling capacity of hydrogels, also known as solvent uptake, determines the amount of water absorbed by the hydrogel, which is fundamental for understanding its behavior in different environments [62,63]. Recent studies have shown that the structure and composition of hydrogels significantly influence their water absorption capacity, highlighting the importance of this characterization in optimizing the design of the gels which will impact drug delivery and the performance of the gels [64,65,66]. The swelling capacity was evaluated by the gravimetric method. Figure 5a,b shows the swelling behavior for the gels PF-Gel-Car and PF-Gel-Sep. PF-Gel-Plu and PF-Gel-Lut were not tested due to their liquid nature which was not suitable for this sort of testing. Carbopol^®^-based gel is the formulation that uptakes more solvent: up to 130 times its dried weight at pH 5.5 and up to 90-fold its dried weight at pH 7.4. This is followed by Sepigel^®^-based gel, which absorbs 40-fold and 30-fold its weight at pH 5.5 and 7.4, respectively. The solvent uptake follows a two-phase profile in which during the first 30 min the gels absorb the solvent fast, following a slower solvent uptake until the gels reach the maximum uptake. The swelling capacity of the gels was higher than the ones prepared by Ahmadi et al., which consisted of pranoprofen loaded in NLC using the same polymers, Carbomer and Sepigel^®^ [43]; this might be due to the lipids from the NLC included in the formulation that may confer lower solvent uptake capacity.

The degradation behavior of hydrogels is one of the factors affecting their suitability for various biomedical applications, including drug delivery systems and tissue engineering scaffolds. Evaluating the degradation of hydrogels using the gravimetric method provides valuable insights into their stability and longevity under physiological conditions [62]. This study aimed to assess the degradation profile of our gel formulations, focusing on the percentage weight loss over time (Figure 5c,d). At the end of the test, Sepigel’s weight was about 25% of the initial gel’s weight at both pHs, and Carbopol’s weight was about 20% at the end of the test.

#### 2.2.6. Evaluation of the Porosity of the Gels

We investigated the porosity of the gels by the solvent displacement method using absolute ethanol as the displacement solvent. PF-Gel-Plu was the formulation that exhibited the highest porosity (93.65 ± 1.93%), which was followed by PF-Gel-Car with a porosity of 88.19 ± 5.98%, PF-Gel-Lut with 85.86 ± 2.69 and finally, PF-Gel-Sep with 82.75 ± 1.17%. The porosity of a gel may impact the swelling behavior in the way that higher porosity usually results in greater swelling [63,67]. This finding was observed in the swelling behavior of the gels where PF-Gel-Car had a superior swelling ratio to PF-Gel-Sep.

### 2.3. In Vitro Release Kinetics of Pranoprofen from Topical Gel Formulations

The release abilities of four gels—Carbomer, Sepigel^®^, Lutrol^®^, and Pluronic^®^—were evaluated using a hyperbola model. As outlined in Table 3, the maximum release capacity (Ymax) and release constant (Kd) were calculated for each gel. Carbomer stood out with the highest Ymax (975.6 µg), meaning it could release the largest amount of substance compared to the other gels. However, it also had the highest Kd (207.6 min), suggesting that pranoprofen was released in a more sustained manner than the rest of the gels. In contrast, Sepigel^®^ had the lowest Kd (11.54 min). Lutrol and Pluronic^®^ fell somewhere in the middle with Lutrol^®^ showing slightly higher drug release.

**Figure 6:**
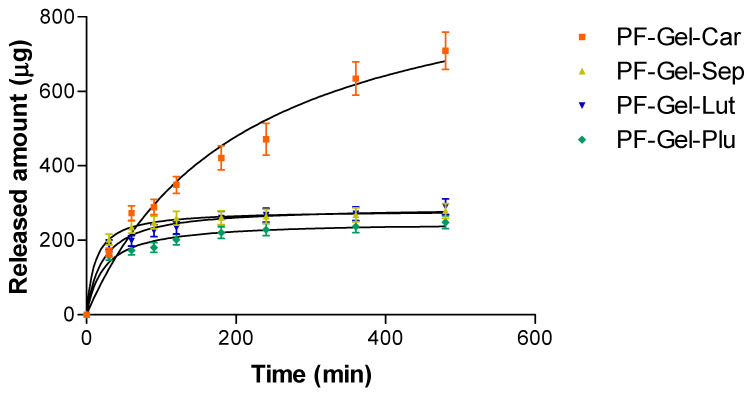
Cumulative release profiles of PF-Gel-Car, PF-Gel-Plu, PF-Gel-Lut, and PF-Gel-Sep over 480 min.

The release of the substance from the gels was tracked over 480 min, as shown in Figure 6. The graph highlights the amount of substance (in μg) that was released over time. Carbomer released the most, hitting nearly 700 μg by the end of the experiment. This aligns with its high Ymax, confirming its ability to release larger quantities. On the flip side, Pluronic^®^ released the least, reaching only around 250 μg, which aligns with its smaller Ymax value predicted by the model. Lutrol and Pluronic^®^ had also low release levels over the same period. The Kd value reflected the speed of release. Carbomer had the highest, meaning it released the substance slower than the rest, offering a more gradual delivery. Sepigel^®^, with the lowest Kd, had the faster release, while Lutrol^®^ and Pluronic^®^ landed in the middle, delivering at a moderate pace (Figure 6) [68,69,70,71].

### 2.4. Ex Vivo Permeation Studies

**Figure 7:**
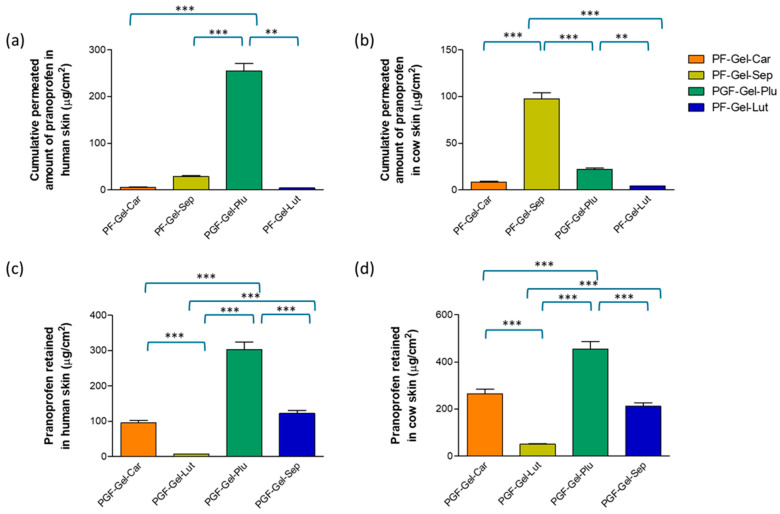
Permeated (**a**,**b**) and extracted (**c**,**d**) amounts of pranoprofen from PF-Gel-Car, PF-Gel-Plu, PF-Gel-Lut, and PF-Gel-Sep on human and cow skin after 24 h of exposure time. ** statistical differences (*p* < 0.001), *** statistical differences (*p* < 0.0001).

An ex vivo permeation study was conducted on human and cow skin to assess the extent of pranoprofen that diffused through the tissues. For a local effect, a limited permeation of the drug is preferred to the systemic circulation, but a high amount of drug is retained in the skin. In this regard, PF-Gel-Plu was the formulation that led to higher PF in cow skin yet restricted the amount in the receptor medium (Figure 7). Additionally, the pH of the gel was about 7, which is a pH value suitable for animal skin.

Concerning the human skin, PF-Gel-Plu formulation led to higher amounts of PF permeating through human skin (about 254 μg/cm^2^, while the amounts of PF permeated for the rest of the gels were below 30 μg/cm^2^). Taking into account the near-neutral pH value of PF-Gel-Plu and the fact that a limited permeation into the receptor fluid is preferred, other gels such as PF-Gel-Car and pF-Gel-Sep would be preferential for human skin because their pH value is close to that of human skin. PF-Gel-Car led to PF being retained in human skin (about 95 μg/cm^2^) and PF-Gel-Sep formulation resulted in a larger amount of PF retained in human skin (about 122 μg/cm^2^).

Previous studies, such as that conducted by Ahmadi et al., have explored the incorporation of nanostructured lipid carriers (NLCs) loaded with pranoprofen (PF) into gels formulated with Carbomer 940 and Sepigel^®^ 305. The ex vivo permeation tests examined transdermal delivery efficacy in semi-solid formulations with nanostructured lipid carriers loaded with pranoprofen (PRA-NLCs) over 24 h duration in a model of human skin in Franz diffusion cells. There was a sustained-release behavior for pranoprofen, and it was therefore concluded that using PRA-NLCs in formulations with gels effectively promotes extended delivery through the dermal tissue. Such extended delivery can maintain the therapeutic concentrations of drugs, and their anti-inflammatory activity can become increased when delivered topically. The authors emphasized the importance of formulation parameters in developing effective NLC-based gels [43]. The results of this previous work are quite in line with ours; Sepigel-based gels loading NLC permeated to a higher extent than Carbomer-based gels with NLC. We observed the same behavior in human skin. However, the amount retained in the skin for PF-Gel-Car and PF-Gel-Sep was similar, whereas Ahmadi et al. observed the opposite: Carbomer-based NLC was the formulation that led to a higher amount of PF in the skin.

Ex vivo permeation experiments are important for testing the transmembrane delivery of drugs in humans and for determining drug absorption and efficacy. Traditionally, these studies have used excised human or animal skin for assessing transdermal drug delivery. However, challenges like limited access to human skin and ethical concerns about animal use have prompted the development of alternatives, such as artificial membranes and three-dimensional cultured human skin models [72]. Choosing the right membrane and model for in vitro and ex vivo permeation studies is crucial. Factors like study objectives, availability, and the drug’s physicochemical properties play a major role. Animal models remain practical for examining percutaneous absorption rates and predicting how topical formulations behave in vivo [73].

### 2.5. Cell Viability Studies

**Figure 8:**
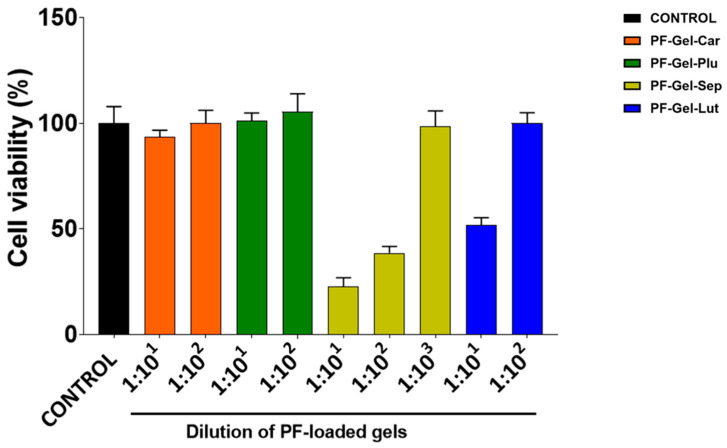
Cytotoxicity effects of the PF-Gels in HaCaT cells.

Cell viability studies performed in HaCaT cells showed that PF-Gel-Car and PF-Gel-Plu were not cytotoxic when tested at dilution of 1:10^1^, as cell viability was close to the untreated control cells (100% cell viability). In contrast, PF-Gel-Sep at dilutions lower than 1:10^2^ caused a significant cytotoxic effect, since cell viability was lower than 50% compared to untreated control cells. For PF-Gel-Lut, the cytotoxicity was around 50% at a dilution of 1:10^1^. PF-Gel-Sep was more cytotoxic than PF-Gel-Lut, since a 10-fold higher dilution was needed for PF-Gel-Sep than for PF-Gel-Lut to reach viability values close to the control cells (Figure 8).

The cytotoxicity analysis in HaCaT cells showed that the two formulations, PF-Gel-Sep and PF-Gel-Lut, required higher dilutions to achieve more than 80% viability. Silva-Abreu and collaborators evaluated the cytotoxicity of Pluronic^®^-based, Carbomer-based, and Sepigel-based gels containing apremilast. They also observed their formulation composed of Pluronic^®^ was the best tolerated [74].

Yet, no irritant effects were observed in the HET-CAM model test for any of these compounds. This discrepancy likely reflects differences in the models. HaCaT assesses direct cellular damage in vitro culture, while HET-CAM evaluates tissue-level responses. The findings suggest that while the product may affect individual cells, it does not cause significant irritation or toxicity in more complex tissues, supporting its potential safety for dermatological use. Anti-inflammatory gels are widely used in dermatology due to their ability to deliver therapeutic agents directly to the skin, providing localized treatment with minimal systemic side effects. Hydrogels, in particular, have gained attention for their ability to provide the sustained release of anti-inflammatory agents, enhancing treatment efficacy and patient compliance [75].

### 2.6. Anti-Inflammatory Efficacy

#### 2.6.1. Anti-Inflammatory Efficacy of PF Gels on Mice

**Figure 9:**
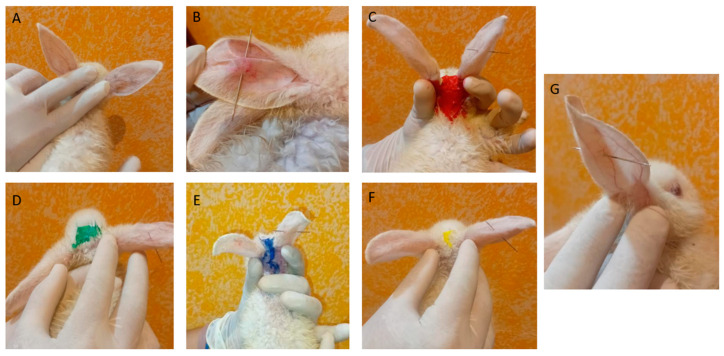
Images of the rabbit ears in the anti-inflammatory efficacy study just after piercing the ears and before applying any treatment. (**A**) Negative control rabbit. (**B**) Positive control rabbit. (**C**) Rabbit before the application of PF-Gel-Car. (**D**) Rabbit before the application of PF-Gel-Plu. (**E**) Rabbit before the application of PF-Gel-Lut. (**F**) Rabbit before the application of PF-Gel-Sep. (**G**) Rabbit before the application of commercial diclofenac as a reference anti-inflammatory product.

The anti-inflammatory efficacy of the gels was assessed on rabbits; for this purpose, their ears were pierced. Figure 9 shows the rabbits on day 1 after the piercing: Figure 9A represents the negative control, where no piercing was performed, and Figure 9B shows the positive control, where a piercing was performed, resulting in redness around the application area. In Figure 9C,D,F, the rabbits were similarly pierced before the application of PF-Gels. Figure 9G shows the piercing of the rabbit to which a commercial diclofenac gel was applied as an anti-inflammatory reference product. In this last figure, due to the closer view, the dilation of blood vessels is noticeable.

**Figure 10:**
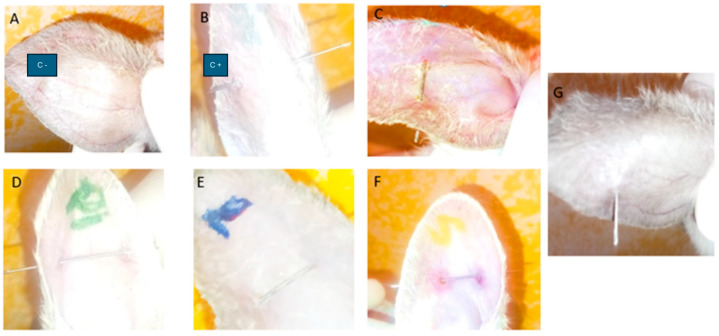
Images of the rabbit ears in the anti-inflammatory efficacy study after 7 days of treatment with PF-Gel after piercing. (**A**) Negative control rabbit. (**B**) Positive control rabbit. (**C**) Rabbit group PF-Gel-Car. (**D**) Rabbit group PF-Gel-Plu. (**E**) Rabbit group PF-Gel-Lut. (**F**) Rabbit group PF-Gel-Sep. (**G**) Rabbit group commercial diclofenac product.

After 7 days of treatment with PF-Gels, no redness was observed in any of the ears of most of the treated groups, except in the PF-Gel-Sep group, where redness was observed around the piercing; it resembled a hematoma and it felt harder to the touch compared to other areas of the ear (Figure 10).

#### 2.6.2. Histological Studies or Microscopic Analysis of Tissue Morphology

**Figure 11:**
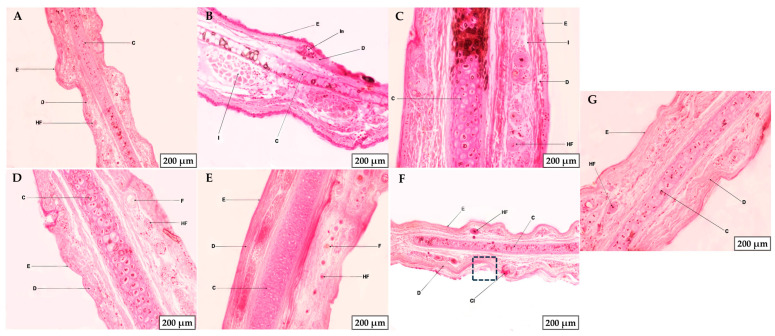
Histological analysis of the skin of rabbit ears subjected to pin perforation, simulating a piercing. (**A**) Negative control. (**B**) Positive control. (**C**) PF-Gel-Car. (**D**) PF-Gel-Plu. (**E**) PF-Gel-Lut. (**F**) PF-Gel-Sep. (**G**) Commercial diclofenac. Scale bar: 200 µm. E: epidermis, D: dermis, I: infiltrates, HF: hair follicles, C: cartilage, F: fat, Cl: clot, In: inflammation.

After the anti-inflammatory efficacy study, a histological analysis of the pierced ears was conducted (Figure 11). The negative control showed intact skin, with healthy epidermis and dermis, showing no damage (Figure 11A). In contrast, the positive control showed inflammatory infiltrates (I) and a portion of the epidermis damaged near an inflamed area of the dermis (In) (Figure 11B). With regard to the treated groups, PF-Gel-Car showed an improvement on the inflammation, with an apparent healthy epidermis, although a few infiltrates were visible (marked as I) (Figure 11C). Conversely, the skin of the rabbit’s ear treated with PF-Gel-Plu was observed as healthy skin with both the epidermis and dermis showing no inflammatory infiltrates or inflammation. For the PF-Gel-Lut, no damage was observed in the epidermis or dermis, showing considerable improvement compared to the other gels. Finally, in the ears treated with PF-Gel-Sep, no inflammation or infiltrates were observed; however, part of the epidermis was slightly damaged (highlighted with a box), and a clot (Cl) was visible within the dermis, corresponding to the area around the puncture site (Figure 11F). In Figure 11G, the control with commercial diclofenac gel shows healthy epidermis and dermis.

### 2.7. The Safety Profile of the Formulations

#### 2.7.1. Tolerance Study by HET-CAM Assay

Since the formulations are intended for being applied to different body areas, including the human face (for instance, eyebrow), we evaluated any possible irritating effects on the eyes by utilizing the HET-CAM technique that involves using the chorioallantoic membrane (CAM) of fertilized chicken eggs, which is a highly vascularized membrane that reacts to irritants in a way similar to human mucous membranes. The egg membranes were evaluated visually by examining for alterations 5 min after applying the PF gels. Results are shown in Figure S1. The irritation score for each formulation was also calculated (Table 4).

The irritation score-based classification is based on the visible bleeding and clotting signs in the CAM monitored for 5 min after applying the formulations. A non-irritant product shows no hemorrhage on the CAM, or the possibility of vessels is minimally affected, similar to the negative control. Contrarily, a severely irritating product shows significant hemorrhaging or vessel lysis. With all the evidence considered, it can be said that the gel formulations tested are not irritating. Major vascular damage or hemorrhaging has not been evident, leading them to be classified as safe for any further use.

#### 2.7.2. Tolerance of the Blank Gels on Human Skin Assessment by Means of the Biochemical Properties of the Skin

**Figure 12:**
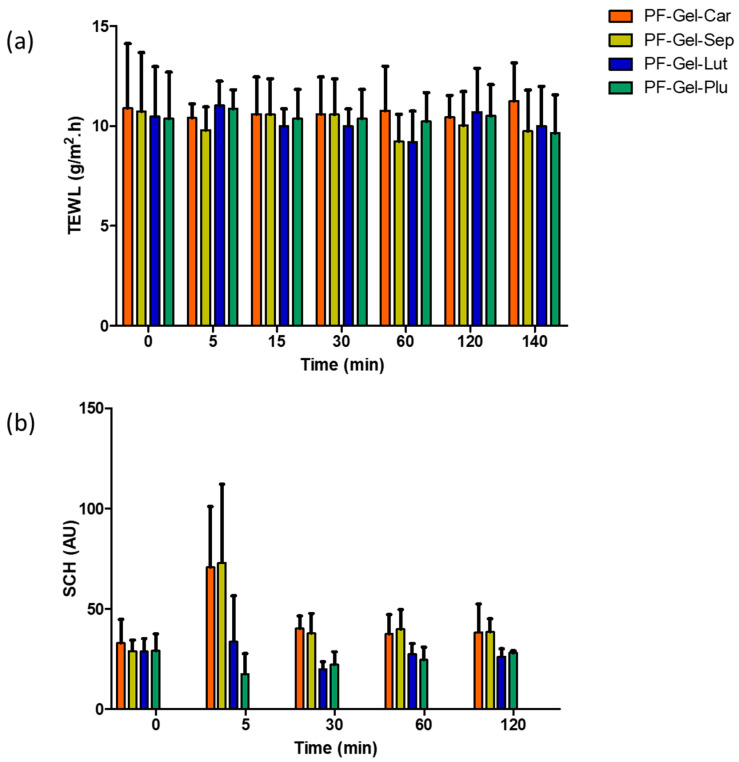
Tolerance studies of the gels on human skin: (**a**) TEWL results over 2 h and 20 min, and (**b**) SCH results over 2 h. The formulations tested were PF-Gel-Car, PF-Gel-Plu, PF-Gel-Lut, PF-Gel-Sep.

Transepidermal water loss quantifies the amount of water that evaporates from the skin, serving as an indicator of skin barrier integrity. Elevated TEWL values typically indicate a weakened skin barrier, which is potentially due to dryness, damage, or various skin conditions [77]. By measuring TEWL, researchers can evaluate the efficacy of gel formulations in restoring or maintaining the skin’s barrier function. Figure 12a presents the results of TEWL for the four blank gels (without pranoprofen) monitored over about 2 h in healthy skin volunteers. No significant statistical changes in TEWL were observed after applying the gels to the skin during the 2 h monitoring period, suggesting that the gel vehicles do not alter the skin barrier function.

Furthermore, proper hydration of the stratum corneum, which is the outermost layer of the skin, is crucial for maintaining skin elasticity and a functional barrier function. Gel formulations are ideally designed to improve skin hydration, so measuring SCH helps determine their effectiveness in providing and retaining moisture. Together, these parameters help evaluate the protective and moisturizing properties of gel formulations, ensuring they are beneficial for skin health [77]. Figure 12b displays the results of SCH for the four blank gels after being applied to the volunteers. The gels composed of Sepigel^®^ and Carbopol^®^ showed an initial increase in skin hydration, which tended to the initial and basal values after 30 min. Contrarily, Pluronic^®^ and Lutrol^®^-based gels caused a slight decrease in hydration—5 min after the application of Pluronic^®^ and 30 min after the application for Lutrol^®^.

## 3. Conclusions

Four different topical gel formulations loaded with pranoprofen (PF) were developed and evaluated for potential use in treating inflammation from piercings and ear tags. The results suggested that the gels exhibited characteristics suitable for topical application and were well tolerated by both the chorioallantoic membrane and human skin. PF-Gel-Car demonstrated superior ability to release the highest amounts of PF and effectiveness in the in vivo anti-inflammation study on the rabbit model; additionally, it exhibited a suitable pH for the human skin. PF-Gel-Plu was found to be highly retained in cow skin, and it showed excellent results in the in vivo anti-inflammatory study, which was also supported by the histological analysis. Furthermore, the pH of the Pluronic^®^-based gel was near a neutral value, which is a suitable pH for animal skin. These topical gels show promise as a viable alternative for managing the inflammation associated with piercings and ear tags. Further research, including in vivo studies and clinical trials, is necessary to validate the efficacy and safety of these formulations in treating inflammation in humans and animals.

## 4. Materials and Methods

### 4.1. Materials

The following substances were employed to create the pranoprofen-loaded gels: PF was supplied by Alcon Cusi (Barcelona, Spain), Sepigel^®^ 305 was purchased from SEPPIC S.A. (Terrasses de l’Arche, 92700 Colombes, France), Pluronic^®^ F-68 was purchased from PanReac AppliChem (Darmstadt, Germany), and Lutrol^®^ F-127 was obtained from BASF ChemTrade GmbH (Burghernheim, Germany). In addition, Carbomer^®^ 940, Nipasol^®^ (propyl 4-hydroxybenzoate), and Nipagin^®^ (methyl 4-hydroxybenzoate) were acquired from Fagron Ibérica (Barcelona, Spain). Triethanolamine 99% was procured from Roig Farma, S.A. (Terrassa, Barcelona, Spain). Transcutol^®^ P was obtained from Gattefossé SAS (Saint-Priest Cedex, France). PBS (phosphate-buffered saline) tablets were purchased from Sigma-Aldrich, (Burlington, MA, USA), prepared following the manufacturer’s instructions, and refrigerated until needed again. The lab-provided MilliQ^®^ Plus System (Darmstadt, Germany) was the source of the filtered water used in all of the studies. An analytical grade was used for all other materials and reagents.

### 4.2. Gels Formulation

First, aqua conservans were prepared as follows: Nipasol (0.022%) and Nipagin (0.05%) were dispersed in Milli Q water (c.s.p. 100 mL). Then, pranoprofen was dissolved in the aqua conservans at a concentration of 1.5%. From this solution, the gels were prepared by adding the gelling agent:

Sepigel^®^ was dissolved in 97 mL of pranoprofen solution and stirred continuously for 30 min to achieve a 3% (*w*/*v*) concentration on a laboratory scale. Additionally, to prepare Gel-Car formulations, carbomer 940 1% (*w*/*v*) was dispersed in the pranoprofen solution (99 mL), and after stirring for 15 min, the formulation was left overnight to swell. The gels were left to rest at room temperature for 24 h to achieve final stability.

Next, in the same way, to prepare 18% (*w*/*w*) Pluronic^®^ and Lutrol^®^, 18 g of each substance was dissolved in 82 mL of pranoprofen solution. This technique made it possible to combine and homogenize the gel samples. The resulting gel formulations were put into glass containers. The resultant solution was kept overnight in the fridge to reach equilibrium.

The next day, the pH of the formulation was adjusted with triethanolamine for the carbomer-based gel and lactic acid for the rest of the gels (see Table 1).

### 4.3. Physicochemical Characterization of the Gels

#### 4.3.1. PH and Morphological Analysis

pH was measured at room temperature with a pH-meter micropH2001 (Crison Instruments SA, Alella, Spain) by triplicate. Gels were dried in a vacuum desiccator to examine their morphology. Following its drying, a tiny amount of each sample was placed on a double-coated carbon conductive tape and coated in an Emitech K950 coater (Quorum Technologies Ltd., Kent, UK) with a thin coating of carbon acting as a conductor agent. With the aid of a JEOL J 7100FE scanning electron microscope (SEM) (Peabody, MA, USA), the inside structure of the gel was investigated [26].

#### 4.3.2. Fourier Transform Infrared (FT-IR) Spectroscopy

Using a Thermo Scientific Nicolet iZ10 with an ATR diamond and DTGS detector, the FT-IR spectra of dried formulations (PF-Gel-Car, PF-Gel-Sep, PF-Gel-Lut and PF-Gel-Plu), and PF were acquired. To obtain dried gels, the formulations were placed in an oven at 40 °C until their weight was constant. The scanning range was 525–4000 cm^−1^ [78].

#### 4.3.3. Rheological Measurements of the Gels

Samples were rheologically characterized using a Haake RheoStress 1 rheometer linked to a Thermo Haake^®^ Phoenix II + Haake C25P temperature control (Thermo Fisher Scientific, Karlsruhe, Germany). It was powered by the Haake RheoWin^®^ Job Manager and Data Manager v. 4.87 software. Steady-state measurements were addressed with a cone and a mobile upper cone (C60/2°Ti: 60 mm diameter, 2° angle). To measure viscosity and flow behavior, each sample was equilibrated for around five min until it reached the operating temperature by being placed between the plate–plate sensor system, 0.5 mm apart [79]. Subsequently, the samples had a three-phase shear profile program consisting of a ramp-up phase (0–50 s^−1^) lasting three min, a constant shear rate period lasting one minute at 50 s^−1^, and a ramp-down period (50–0 s^−1^) lasting three min. The tests were conducted at 25 °C 24 h after the gels were made [74].

Several mathematical models, including Newton, Bingham, Ostwald-de-Waele, Herschel–Bulkley, Casson, and Cross, were fitted to the data from the flow curves (shear stress (τ) vs. shear rate (Y). The best correlation coefficient value (r) was used to pick the model that statistically best described the experimental data [80].

#### 4.3.4. Spreadability Test

The approach previously outlined by Sanz et al. served as the foundation for the extensibility test. The high spreadability of topical preparations is also a determinant of their medicinal effectiveness. To assess this feature, equal amounts of samples were arranged in a circle that had been previously drawn on a glass plate. A second glass plate was then put over the first one, as centrally as possible, to avoid the plate sliding. Weights of 0, 1, 2, 5, 10, 20, 50, and 100 g were added to the upper plate to exert force and compress the sample until it reached a consistent thickness. After 60 s, the weights were removed and the area of the sample was measured. Each sample was analyzed three times for each weight at room temperature. The results were explained in terms of the spreading area as a function of the applied mass using the following equation:

(1) $$S=d^{2}\times \pi /4$$

where *S* is the spreading area (cm^2^) that the applied mass (g) produced and *d* is the sample’s mean diameter (cm) [79,81].

#### 4.3.5. Swelling and Degradation Tests

Dried gel samples were used to evaluate the swelling capacity of the gels. Firstly, the gels were dried by placing them in an oven at 40 °C until constant weight. Then, a weighed amount of dried gel was placed in a non-waved bag which was immersed in PBS buffer (pH 5.5 and 7.5) in an incubator set to 32 °C, and they were collected and weighted after removing excess PBS at the time points of 5, 25, 60, 80 and 95 min. The swelling test assesses how effectively gels can hold onto water inside of their structure. Using Equation (2), the swelling ratio (*SR*) was calculated using a gravimetric technique to determine the PBS uptake including 4 replicates for each gel:

(2) $$SR=\frac{W_{s}-W_{d}}{W_{d}}$$

where *Ws* is the weight of the gels that have swelled at various periods, and *W_d_* is the weight of the gels that have dried.

In summary, after taking the gel out of the incubator, the extra PBS was soaked up and weighed (*Ws*) [82,83].

The percentage of weight loss (*WL*) was used to assess the gel breakdown rate (degradation). Similar to the swelling, PBS (at two pHs, 5.5 and 7.5) was used to submerge known volumes of gels at 32 °C. Next, samples (*n* = 4 for each gel) were weighed at predetermined intervals of time after blotting the bags. The *WL* was determined using the following Equation (3):

(3) $$WL\left(\%\right)=\frac{W_{dt}}{W_{i}}100\%$$

The weight of the gels at different intervals is shown by *W_dt_*, while their beginning weight is indicated by *W_i_*.

#### 4.3.6. Evaluation of the Porosity of the Gels

The porosity of the gels was determined by the solvent displacement method which consisted of immersing weighed amounts of dried gel in absolute ethanol. The gels’ weight was measured after removing the excess ethanol. To determine the porosity of the gel, the following equation was used (Equation (4))

(4) $$\%\;porosity=\frac{Wf-Wi}{\rho \times V}\times 100$$

where *Wf* is the final weight of the gel, *Wi* is the initial weight of the gel, *ρ* is the density of the absolute ethanol and *V* is the volume of the gel.

#### 4.3.7. In Vitro Release Kinetics of Pranoprofen from Topical Gel Formulations

Drug release tests were conducted utilizing Franz-type diffusion cells (FDC 400, Crown Glass, Somerville, NY, USA) to evaluate the release profiles of pranoprofen from four different topical gel formulations [84]. For each test, 0.1 g of the gel formulation was placed inside the donor compartment of the Franz cells.

The receptor compartment contained 4 mL of a solution of 5% Transcutol^®^ and Milli-Q water, which was maintained under constant stirring throughout the experiment. Samples (0.5 mL) were withdrawn at predetermined time intervals (30 min, 1, 1 h 30 min, 2 h, 3 h, 4, 6, and 8 h) and replaced with an equal volume of fresh Transcutol^®^ 5% and MilliQ water to maintain sink conditions. The experimental conditions are summarized in Table 5.

The experiment was conducted for a total duration of 8 h (480 min). The concentration of pranoprofen in the receptor fluid was determined using high-performance liquid chromatography (HPLC). The detailed experimental conditions for the release study are summarized in the table below:

**Table 5:** Description of the conditions applied during the in vitro release experiments.

Parameter	Condition
Receptor fluid	Transcutol 5% + Milli Q water
Cell volume	5 mL
Membrane	Nylon membrane
Diffusion area	0.64 cm^2^
Temperature	32 ± 0.5 °C
Stirring	600 r.p.m.
Dose	0.1 g
Sample volume	0.5 mL
Sampling times	0, 30 (min), 1, 1.5, 2, 3, 4, 6, and 8 (h)
Replicates	*n* = 3

In the in vitro drug release study, various kinetic models were applied to the experimental data using nonlinear regression analysis. The goodness of fit for each model was assessed by calculating the determination coefficient (r^2^) with the model exhibiting the highest r^2^ value considered the best fit [85,86].

### 4.4. Ex Vivo Permeation Studies

#### 4.4.1. Biological Tissues

Human skin tissue: From abdominoplasties performed on healthy women, dermatomed human skin was acquired (Barcelona SCIAS Hospital, Barcelona, Spain). The skin was sliced into sheets that were 400 ± 50 μm thick. The study protocol (Nº002; authorized on 17 January 2020) was accepted by the Bioethics Committee of the Barcelona-SCIAS Hospital, and informed consent forms were supplied in writing by the participants. The skin was refrigerated at −20 °C after the procedure until the studies were conducted. Using microneedles to puncture the epidermis, the skin was physically altered to resemble AD skin.

Cow skin tissue: The Animal Facility (Bellvitge Campus, University of Barcelona, Spain) provided us with cow skin. The test was conducted using a 500 µm thick dermatomized layer (GA630, Aesculap, Tuttlingen, Germany). The study protocol was approved by the University of Barcelona’s Ethics Committee for Animal Experimentation on 10 January 2019.

#### 4.4.2. Ex Vivo Evaluation of Skin Permeability

The European Medicines Agency (EMA), the Food and Drug Administration (FDA) of the United States, and the Organization for Economic Co-operation and Development (OECD) all endorse Franz diffusion cells as a common analytical setup for assessing skin absorption in vitro. PBS phosphate-buffered (pH = 7.4) saline with 5% Transcutol^®^ was used as the receptor fluid in these 5 mL Franz diffusion cells with a permeation area of 0.64 cm^2^, which were set up manually and vertically [87]. A water bath that was heated to 32 °C was used to hold the Franz diffusion cells. The donor compartments were positioned on top of the skin, and the dermatomed skin circles were placed on top of the acceptor compartment. At a speed of 600 rpm, the receptor fluid was stirred there. At precise intervals, 0.2 mL of the samples was removed from the receptor chamber and replaced with the same volume of receptor media. We used a validated HPLC-UV technique to analyze the samples. The detailed experimental conditions and results are summarized in Table 6, which provides a comprehensive overview of the ex vivo skin permeation test setup and key parameters.

#### 4.4.3. Analytical Method by HPLC

High-performance liquid chromatography (HPLC) analysis of the samples from the drug release and a permeation test of the four gels (PF-Gel-Car, PF-Gel-Plu, PF-Gel-Lut, and PF-Gel-Sep) was conducted in this study. The analysis was conducted with a Waters 2695 Separations Module and a Waters 2996 Photodiode Array (PDA) Detector, which is a system with a high level of accuracy in compound analysis. Optimized chromatographic conditions, specific for the effective determination and detection of compounds in samples from PF-Gels, were programmed in the system. Detailed chromatographic conditions are summarized in Table 7.

### 4.5. Cell Viability Studies

Cell viability was evaluated using the MTT (3-(4,5-Dimethylthiazol-2-yl)-2,5-Diphenyltetrazolium Bromide) assay in human keratinocyte (HaCaT) cells, utilizing the reduction of tetrazolium salt as the readout. The cells were seeded at a density of 2 × 10^4^ cells per well in 96-well plates containing 100 μL of DMEM culture medium supplemented with 10% fetal bovine serum (FBS), 100 U/mL penicillin G, 100 µg/mL streptomycin, and 2 mM L-glutamine. Cells were incubated at 37 °C in a humidified 5% CO_2_ atmosphere until reaching 80–90% confluence. For the cytotoxicity analysis, 10 mg of each of the PF-Gels under study were diluted in 1 mL of DMEM; afterward, a series of dilutions of PF-Gel-Car, PF-Gel-Plu, PF-Gel-Sep, and PF-Gel-Lut were prepared in DMEM and added to the cell culture. After 48 h of incubation, the medium was removed, and MTT (0.25% in PBS; Sigma-Aldrich, St. Louis, MO, USA) was added. Following 3 h of incubation, the MTT solution was replaced with 100 µL of DMSO (99%, Sigma-Aldrich). Absorbance was measured at 570 nm using a Modulus^®^ Microplate Photometer (Turner BioSystems Inc., Sunnyvale, CA, USA). Cell viability was expressed as percentage relative to untreated control cells and calculated using the following formula: Cell viability = (Abs treated cells/Abs control cells) × 100.

### 4.6. Anti-Inflammatory Efficacy Study of PRA on Rabbit Ears

#### 4.6.1. Anti-Inflammatory Efficacy

For testing the anti-inflammatory efficacy of the gels, albino New Zealand rabbits were used. The study was performed in accordance with the guidelines provided by CENBIO-UNAH, and the study protocol was approved by the Ethics Committee of CENBIO-UNAH (protocol code CICUAL 003-2025 approved on 14 March 2025). Rabbits weighing 1.5 kg were acclimatized for a period of 5 days prior to the study and were classified into 7 groups (*n* = 5/group): Group A (negative control), Group B (positive control), Group C (PF-Gel-Car), Group D (PF-Gel-Plu), Group E (PF-Gel-Lut), Group F (PF-Gel-Sep), and finally Group G (commercial diclofenac gel). The ears were cleaned with chlorhexidine and, after 10 min, they were pierced with a previously sterilized pin (a double piercing simulating the application of piercings as in cases of nipple or eyebrow piercings). Subsequently, 0.5 g of each gel under study was applied every 12 h for 7 days. On the eighth day, the rabbits were anesthetized and euthanized with sodium pentobarbital, adhering to the 3Rs principles.

#### 4.6.2. Histological Studies or Microscopic Analysis of Tissue Morphology

Thereafter, a histological analysis of the rabbits’ ears was performed. Briefly, the pins were removed, and after the euthanasia process, the rabbits’ ears were cut and washed with sterile distilled water. They were fixed for 24 h in Orth-ER liquid fixative (5 g potassium dichromate, 5 mL glacial acetic acid, 5 mL commercial formalin (MERK, Darmstadt, Germany), and 90 mL distilled water). Subsequently, the excess fixative was removed with constant running water for 4 h. To dehydrate without damaging the tissues, the samples were immersed in a gradual series of ethanol solutions at different concentrations (50%, 60%, 70%, 80%, 90%, 95%, and 99%) for an average of 6 h at each concentration (DIMELAB, Tegucigalpa, Honduras). All samples were embedded in paraffin blocks, sectioned into 10 µm slices, and mounted on slides. The samples were then stained with hematoxylin and eosin and finally observed under a microscope (Olympus CX31, Tokyo, Japan) equipped with a camera at 100× magnification to evaluate the tissue structure in a blinded manner.

### 4.7. The Safety Profile of the Formulations

#### 4.7.1. Tolerance Study by HET-CAM (Irritancy Assessment)

The Hen’s Egg Test–Chorioallantoic Membrane (HET-CAM) assay was used to evaluate the irritant potential of the gels developed. Fertilized hen eggs (10 days) were obtained from the G.A.L.L.S.A. farm (Tarragona, Spain). On day 10 of incubation, a small window was created in the eggshell to access the CAM. About 300 mg of gel was applied directly onto the CAM. A control group was treated with NaOH 0.1 N, which was used as the positive control, and for the negative control eggs, a solution of 0.9% NaCl was applied. After gel application, the effects were recorded for 5 min, focusing on the onset of hemorrhage, coagulation, and vessel lysis, if any. The degree of irritation was scored based on a standardized scale [76] with higher scores indicating greater irritant potential.

IS = 301 − sec H × 300⋅5 + 301 − sec L × 300⋅7 + 301 − sec C × 300⋅9(5)

where H is hemorrhage, L is vessel lysis, C is coagulation, and sec is the time in seconds when signs started.

#### 4.7.2. Tolerance of the Blank Gels on Human Skin Assessment by Means of the Biochemical Properties of the Skin

The skin barrier’s physical layer is measured using the most widely utilized techniques. The most often addressed metrics are transepidermal water loss (TEWL) and stratum corneum hydration (SCH). While TEWL measures dynamic water losses, SCH evaluates the skin’s static water-holding capacity. Studies suggest that employing both of these measurements, which are often used together, may be required to completely describe how the skin barrier functions. Moreover, since the forearm is a readily accessible region frequently utilized in dermatological research, it allows for easier comparisons with results from other investigations [88]. Ten female volunteers with healthy skin participated in the study. The volunteers’ age ranged 25–45 and their skin phototype was III–IV.

- A common method for evaluating and quantifying skin-insensible water loss is transepidermal water loss (TEWL), which is frequently used to evaluate the integrity of the skin’s barrier function. The TEWL was measured by a basic device called a Tewameter^®^ (TM 300 Courage-Khazaka Electronics GmbH, Cologne, Germany). The majority of measuring techniques make use of skin contact equipment. The room temperature was 25 °C, and participants were given 15 min to acclimatize to the room’s conditions and allow their skin to reach equilibrium and adapt to environmental conditions before taking measurements. Additionally, participants were requested to refrain from applying topical lotions or oils to the areas of the skin that would be assessed before the measurement process [89]. After that, the skin was covered evenly with around 0.5 g of PF-Gel-Plu, PF-Gel-Lut, PF-Gel-Car, and PF-Gel-Sep. Measurements were made at time intervals (0, 5, 15, 30 min; and 1, 2, 2:20 h). For sixty seconds, the electrode was placed on the skin’s surface without applying any pressure. Low TEWL values often indicate a functioning barrier that is intact on the skin, whereas an elevated TEWL indicates a skin barrier that is damaged or disrupted. The results were presented as mean ± standard deviation (*n* = 4) and represented as g/m^2^/h for transepidermal water loss (TEWL).
- Water content in the stratum corneum is measured by SCH. Using an 825 Corneometer^®^ (Courage & Khazaka Electronics GmbH, Cologne, Germany), the stratum corneum’s (SCH) level of hydration was assessed. At intervals of 0 min, 5 min, 15 min, 30 min, 1 h, and 2 h, measurements were taken, and the number of selected candidates was equal to the TEWEL test. Additionally, the capacitance method was used to conduct the tests.

## Figures and Tables

**Table 1:** pH of the gels 24 h after their preparation.

Sample	pH
PF-GEL-LUT	7.67 ± 0.02
PF-GEL-CAR	5.39 ± 0.01
PF-GEL-PLU	7.16 ± 0.01
PF-GEL-SEP	6.23 ± 0.02

**Table 3:** Release parameters (Ymax and Kd) and confidence intervals for PF-Gel-Car, PF-Gel-Plu, PF-Gel-Lut, and PF-Gel-Sep.

Hyperbola (Y = Ymax × X/Kd + X)	PF-Gel-Car	PF-Gel-Sep	PF-Gel-Lut	PF-Gel-Plu
** Best-fit values**				
Ymax (µg)	975.9	280.1	288.6	249.0
Kd (min)	207.9	11.54	21.34	23.24
** Std. Error**				
Ymax	94.56	1.968	8.396	7.904
Kd	43.38	0.7248	3.756	4.250
95% CI (profile likelihood)				
Ymax	752.3 to 1200	275.5 to 284.8	268.7 to 308.5	230.3 to 267.7
Kd	105.3 to 310.4	9.827 to 13.26	12.45 to 30.22	13.19 to 33.29
** Goodness of Fit**				
Degrees of Freedom	7	7	7	7
R squared	0.9918	0.9996	0.9946	0.9939
Sum of Squares	3229	23.4	328	277.4
Sy.x	21.48	1.828	6.846	6.295

Ymax is the maximum drug release predicted by the model; Kd is the time at which the drug release reaches the 50%.

**Table 4:** Irritation classification of the tested formulations of gels based on HET- CAM results.

Formulations	Irritation Score (IS)	Classification
PF-Gel-Plu	0.1	Non-irritating
PF-Gel-Car	0.1	Non- irritating
PF-Gel-Sep	0.1	Non-irritating
PF-Gel-Lut	0.1	Non-irritating

Irritation Classification: non-irritating (score < 0.9) [76].

**Table 6:** Detailed protocol for ex vivo permeation testing of topical formulations.

Parameter	Condition
Receptor fluid	Phosphate buffered saline (PBS pH = 7.4), Transcutol^®^ 5%
Cell volume	5 mL
Membrane	Abdominal human skin, Cow breast skin
Diffusion area	0.64 cm^2^
Thickness	0.4 mm (Human), 0.7 mm (Cow)
Temperature	32 ± 0.5 °C
Stirring	600 r.p.m.
Dose	0.2 g
Sample volume	0.2 mL
Sampling times	24 h
Replicates	*n* = 5

**Table 7:** Optimized chromatographic conditions for the HPLC analysis of PF-Gel formulations, including PF-Gel-Car, PF-Gel-Plu, PF-Gel-Lut, and PF-Gel-Sep.

Parameter	Condition
Chromatographic column	Kromasil 100 C18 (15 × 0.46 mm, 5 µm)
Mobile phase	Methanol: Acetic 1% (A: B)
Flux	0.8 mL
Pump mode	Gradient
	From 0–10 min: 55% A: 45% B 10–11 min: 20% A: 80% B 11–15 min: 55% A: 45% B
Injection volume	20.00 µL
Run time	15:00 min
Wavelength	254.0 nm

## Data Availability

The data presented in this study are available on request from the corresponding author.

## Supplementary Materials

The following supporting information can be downloaded at https://www.mdpi.com/article/10.3390/gels11050334/s1, Figure S1: HET-CAM test: Results of the potential irritation effects of the four gels on CAM of fertilized chicken eggs. Upper panels show the CAM before applying the formulations and lower panels at 5 min post-application: C− (saline solution), C+ (sodium hydroxide solution 0.1 N, PF-Gel-Car, PF-Gel-Plu, PF-Gel-Lut, PF-Gel-Sep.
