# Supplementary material for: Novel Gels for Post-Piercing Care: Evaluating the Efficacy of Pranoprofen Formulations in Reducing Inflammation
Source: Gels. 2025 Apr 30;11(5):334. doi: 10.3390/gels11050334 (PMC12110840; doi:10.3390/gels11050334)
Supplement: Supplementary file 1 [file gels-11-00334-s001.zip › gels-3511019-supplementary.pdf]

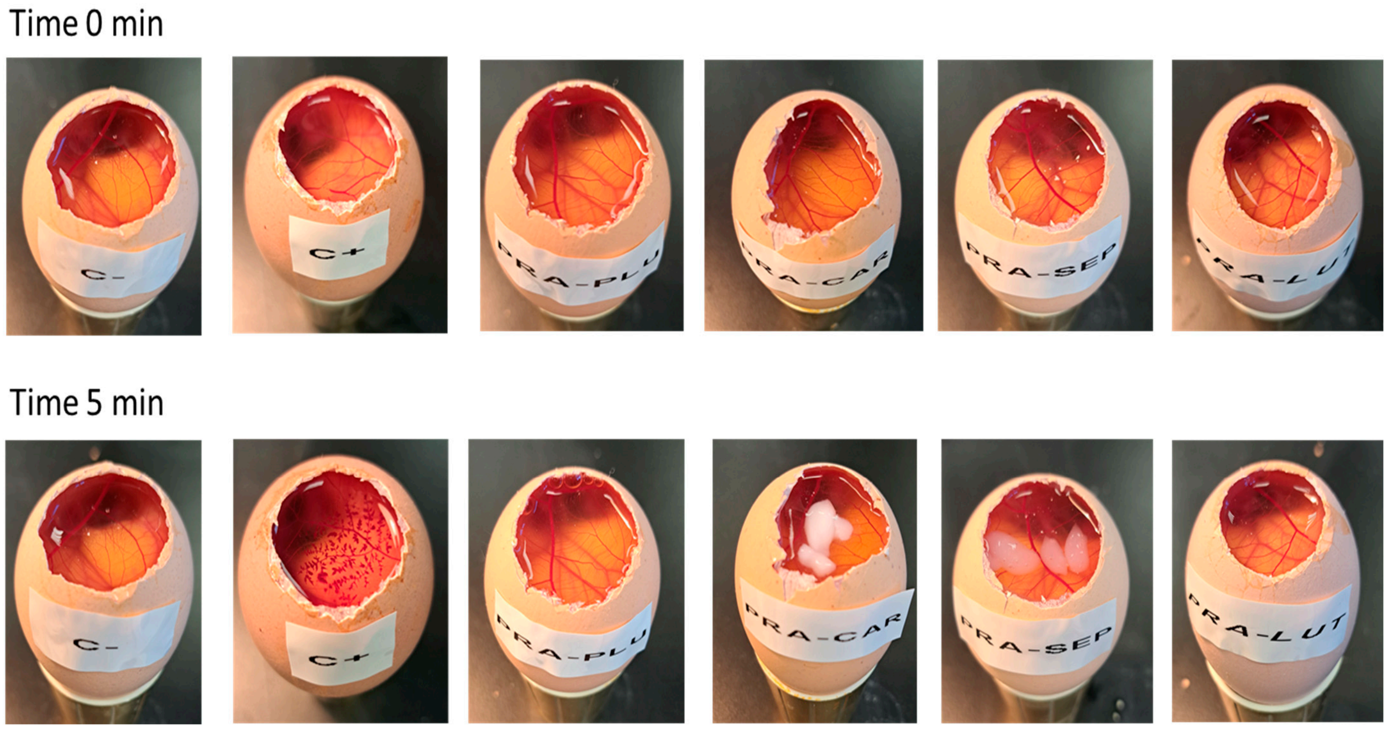

Figure S1. HET-CAM test: Results of the potential irritation effects of the four gels on CAM of fertilized chicken eggs. Upper panels show the CAM before applying the formulations and lower panels at 5 minutes post-application: C- (Saline solution), C+ (sodium hydroxide solution 0.1 N, PF-Gel-Car, PF-Gel-Plu, PF-Gel-Lut, PF-Gel-Sep.
